# Diabetes Insipidus as an Early Clinical Indicator of Wolfram Syndrome Type 1: Evidence From a Symptom-Based Screening Approach

**DOI:** 10.1155/pedi/8692152

**Published:** 2025-10-02

**Authors:** Ozge Beyza Gundogdu Ogutlu, Atilla Cayır, Ayşe Sena Donmez, Serkan Bilge Koca, Oguzhan Yarali, Huseyin Demirbilek

**Affiliations:** ^1^Department of Medical Genetics, Erzurum Regional Training and Research Hospital, Erzurum, Türkiye; ^2^Department of Pediatric Endocrinology, Ataturk University Faculty of Medicine, Erzurum, Türkiye; ^3^Department of Pediatrics, Erzurum Regional Training and Research Hospital, Erzurum, Türkiye; ^4^Department of Pediatric Endocrinology, University of Health Sciences Kayseri City Educational and Research Hospital, Kayseri, Türkiye; ^5^Department of Pediatric Endocrinology, Hacettepe University Faculty of Medicine, Ankara, Türkiye

**Keywords:** diabetes insipidus, early detection, genetic testing, wolfram syndrome type 1, WS1

## Abstract

**Objective:** Wolfram Syndrome Type 1 (WS1) is a rare neurodegenerative disorder characterized by diabetes insipidus (DI), diabetes mellitus (DM), optic atrophy (OA), and deafness (D) due to biallelic mutations in the *WFS1* gene. As the cardinal symptoms of DI, polyuria and polydipsia, overlap with those of DM, DI might be underdiagnosed or delayed in the early stages of WS1. In the present study, we assessed whether DI could be an early sign of WS1 and analyzed genotype–phenotype correlations in a group of Turkish patients with Type 1 DM.

**Patients and Methods:** We applied a polyuria/polydipsia questionnaire to 1278 children with Type 1 DM. Patients with suggestive symptoms of DI were further evaluated for other clinical features of WS1 and molecular genetic analysis of the *WFS1* gene. Clinical, laboratory, and genetic characteristics of cases identified using questionnaires were compared with a historical case series of seven children with WS1 and previously published literature data.

**Results:** Eighteen patients were considered to have a diagnosis of DI, thereby being eligible for genetic analysis of *WFS1* variants. Of those, six had biallelic variations (four missense variants, one in-frame duplication, and three frameshift variants) in the *WFS1* gene, and a diagnosis of WS1 was confirmed. The age of admission for DM was younger in the historical cases (5.1 ± 2.0 vs. 8.7 ± 3.4; *p*=0.04). There was no statistically significant difference between the ages for the diagnosis of WS1 (12.9 ± 5.0 vs. 9.6 ± 2.7; *p*=0.191), though the diagnostic delay from DM onset to WS1 diagnosis was significantly shorter in the screened group (median 1.8 vs. 6.9 years; *p* ≈ 0.015).

**Conclusion:** Our findings suggest that DI may present before OA in WS1. Enriching the diagnosis of DI using a simple polyuria/polydipsia questionnaire may provide an earlier diagnosis of WS1 in patients followed with Type 1 DM. Screening and early genetic testing of these patients enhances the diagnosis, follow-up, and management strategies of patients with WS1.

## 1. Introduction

Wolfram Syndrome Type 1 (WS1), a rare autosomal recessive neurodegenerative disorder characterized by diabetes mellitus (DM), diabetes insipidus (DI), optic atrophy (OA), and deafness (D) [1, 2]. Its estimated prevalence is between 1 in 100,000 and 1 in 770,000 worldwide [2, 3]. Biallelic recessive variants in the *WFS1* gene, which encodes for wolframin protein, account for the underlying molecular genetics etiology of WS1 [4]. *WFS1* mutations result in protein misfolding and increased ER stress [5, 6].

More than 200 different mutations have been described in the literature, as highlighted by recent reviews on the genetic landscape of WS1 [6]. However, a search of the ClinVar database reveals an even higher number, with 281 reported *WFS1* variants [7]. Over time, several studies have proposed genotype–phenotype classification systems to understand the variable clinical course of WS1 better. Rigoli et al. [8] first categorized *WFS1* mutations into three groups based on mutation type, noting that patients with nonsense or frameshift mutations had earlier onset of DM, DI, and hearing loss (HL), though no difference was observed in OA onset. Rohayem et al. [9] introduced a function-based classification system that grouped patients according to the predicted severity of their *WFS1* variants, highlighting that more deleterious genotypes were associated with earlier disease onset. This approach was later adopted and expanded by de Heredia et al. [10], who refined the system into five classes (A1, A2, A3, B, C). Their large-scale meta-analysis revealed that patients in Class A1 were associated with earlier DM onset, those in Class A2 with earlier OA and DI onset, and Class C patients tended to experience earlier onset of multiple clinical features and a faster disease progression compared to the general WS1 population [10].

Although the minimal diagnostic threshold is coexisting early-onset DM and OA approximately 50% of affected individuals will ultimately develop other features of WS1, including DI and HL [11]. A systematic review by de Heredia et al. [10], which covered 15 years and included data from 412 WS1 patients, revealed the chronological onset of major symptoms. DM usually appears in the first decade, followed by OA in the early second decade, and DI and HL typically develop in the second decade. In the third decade, other complications, such as urinary tract dysfunction and neurological disorders, emerge. However, the review also found a high level of variability between the presentation of symptoms, as some patients presented with OA or HL before DM. The study further assessed whether relying on currently used criteria, in which both the DM and OA are required, is sufficient for identifying all cases of WS1. Defining juvenile onset as occurring at 18 years or younger, it was discovered that 14.87% (47 of 316) of WS1 patients do not have both DM and OA, indicating that the current criteria might fail to achieve the diagnosis of WS1 patients [10].

Polyuria and polydipsia are cardinal symptoms for two of the core diagnostic features of WS1 (DM and DI). Therefore, overlapping this symptom between DM and DI may result in a delayed or underdiagnosis of DI in the early stages of WS1. According to the best practice guide written by Mahon et al. [12] for evaluating children with polyuria and polydipsia, it is essential to ask specific questions to assess the presence of these symptoms accurately (Table 1).

In the present study, we assessed whether DI could be an early sign of WS1 and analyzed genotype–phenotype correlations in a group of Turkish patients with Type 1 DM compared to a historical case series of patients with the diagnosis of WS1.

## 2. Patients and Methods

### 2.1. Patient Selection

The initial study population included 1936 pediatric patients diagnosed with Type 1 DM between 2015 and 2023 at the Pediatric Endocrinology Clinic of Erzurum City Hospital. Among these, 1278 patients who consistently attended regular follow-up visits were included in the study. Autoantibody testing (anti-GAD, anti-islet cell, and anti-insulin antibodies) was performed shortly after diabetes diagnosis for all patients. Of these, 182 patients (14.3%) tested negative for all three autoantibodies. Due to limited laboratory facilities, more sensitive autoantibodies, IA2 and ZnT8, testing was not applicable. Given the high risk of false–negative autoantibody testing and our primary goal of early detection of DI as a cardinal diagnostic feature of WS1, we administered the polyuria/polydipsia questionnaire to all 1271 cases (Table 1). According to the data from the questionnaire, 18 patients were considered to have polyuria/polydipsia and the diagnosis of DI was confirmed by a standard water deprivation test. Genetic analysis was conducted only in cases with a diagnosis of DI and negative autoantibody results. Of those, six had biallelic variations in the *WFS1* gene, and a diagnosis of WS1 was confirmed. All patients with biallelic *WFS1* variants were evaluated for additional clinical features of WS1, including OA, HL, and other recognized WS1-related complications. These newly identified cases were compared with the historical WS1 cohort from our center and with cases reported in the literature. Seven patients who had already been diagnosed with WS1, confirmed by genetic testing, were designated as the historical WS1 cohort. Our team had previously reported two patients from the historical WS1 cohort (C1/F1 and C2/F1) [13].

**Figure 1:**
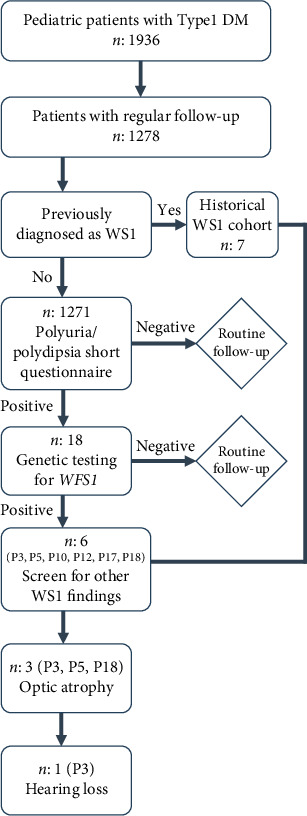
Flow diagram showing the patient selection process for those who underwent genetic testing for *WFS1* variants, indicative of WS1.

The Erzurum City Hospital Ethical Committee approved this study with the ethical approval number BAEK-2024/12-2014.  Figure 1 displays the workflow for patient selection, follow-up, screening, genetic testing of *WFS1* variants, and follow-up clinical evaluations.

Large language model-based tools (e.g., AI writing assistants) were used to support language editing. No AI-generated content was used in data analysis, interpretation, or content generation. All outputs were critically reviewed and verified by the authors.

### 2.2. Molecular Genetic Analysis

The genomic DNA was extracted from peripheral blood samples (200 µL) using the Qiagen QIAamp DNA Blood Mini QIAcube Kit (Qiagen, Hilden, Germany) according to the manufacturer's instructions. The *WFS1* gene was sequenced by the next-generation sequencing technique using the Nextera XT DNA Library Prep Kit on the Illumina MiSeq system (Illumina Inc., USA) according to the manufacturer's protocols. Variants were filtered using the Variant Annotation and Filtration Tool (VarAFT), and classified according to the American College of Medical Genetics (ACMG/AMP) 2015 guideline and the ACGS 2019 guideline [14, 15]. Population frequency was obtained from the Genome Aggregation Database (GnomAD). We used in silico tools such as Revel, MetaLR, and Gerp++ to assess missense variants.

### 2.3. Genotype–Phenotype Correlation

To assess genotype–phenotype correlations, all biallelic *WFS1* variants identified in this study were classified according to three established frameworks proposed by Rigoli et al. [8], Rohayem et al. [9], and de Heredia et al. [10]. These systems categorize mutations based on type (e.g., missense and frameshift), predicted impact on protein function and allelic combination patterns. The resulting classifications were then compared with clinical data—including age of onset for DM, OA, DI, and HL—to explore possible associations. Intrafamilial variability and mutation-specific trends were also evaluated.

## 3. Results

Of the 1278 pediatric patients with diabetes who were regularly followed in the pediatric endocrine clinic, seven had previously been diagnosed with WS1. These patients were not included in the polyuria/polydipsia screening process and were designated as the historical WS1 group. The remaining 1271 patients exhibited no clinical signs of OA or other WS1-related complications during routine follow-up visits and were screened using a polyuria/polydipsia questionnaire to detect possible early symptoms of DI.

Based on questionnaire responses, 18 patients from 17 families who were also negative for diabetes-related autoantibodies were selected for further analysis and underwent *WFS1* gene sequencing. Of these, six patients (four females) were found to carry biallelic variants (four missense variants, one in-frame duplication, and three frameshift variants) in the *WFS1* gene, and a diagnosis of WS1 was confirmed. The median age at WS1 diagnosis in this group was 9.5 years (range: 6.17 years to 17 years). All six genetically confirmed WS1 patients underwent further evaluation for associated clinical features. OA was identified in three patients (P3, P5, and P18), diagnosed at 11.17, 11.5, and 12.92 years of age, respectively. Sensorineural HL was present in only one patient (P3), diagnosed at 8 years of age.

**Figure 2:**
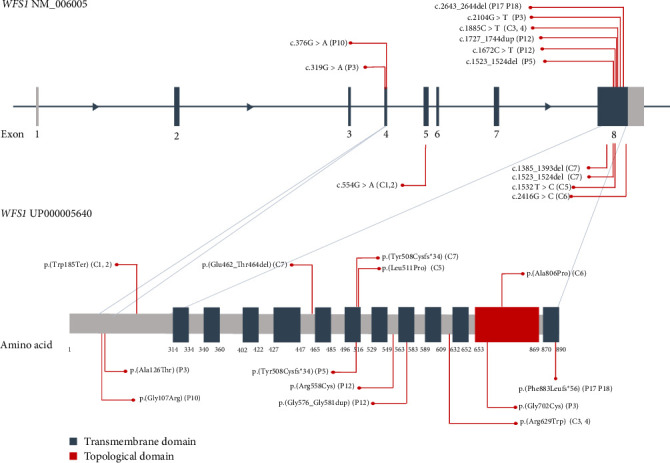
Schematic representation of the *WFS1* gene and protein structure, illustrating the distribution of identified variants in patients with WS1. The upper panel displays the exon–intron structure of the *WFS1* gene, while the lower panel represents the corresponding wolframin protein structure and its domains. Variants identified in patients who presented with DI as the initial symptom following DM are shown in red at the top of the gene diagram and the bottom of the protein diagram. Conversely, variants from patients in whom OA or HL followed DM are displayed in red at the bottom of the gene diagram and the top of the protein diagram. Variants include those identified in the newly screened and historical WS1 cohorts. *⁣*^*∗*^ represents a stop codon, indicating that the variant causes a premature termination of protein translation (nonsense mutation), according to HGVS nomenclature.

Genetic analysis revealed seven distinct *WFS1* variants among the six newly identified patients, comprising four missense mutations, one in-frame duplication, and two frameshift variations. Five of these variants had previously been reported in the literature, while two were novel. The distribution of these variants is illustrated in Figure 2, which depicts their positions within the *WFS1* gene and corresponding protein domains.

A comparative assessment was conducted between patients who exhibited early-onset DI and those in whom other features, such as OA or HL, appeared first. This included the six newly diagnosed patients and two historical siblings from Family 2 (C3 and C4), all of whom presented with DI as an initial symptom following DM. These cases were contrasted with the remaining historical patients whose first features were OA or HL. No variant clustering was observed in specific protein domains among early-DI patients compared to others, suggesting a lack of domain-specific correlation with DI onset.

Notably, patient P5 and patient C7 both carried the same frameshift variant (c.1523_1524del), yet presented differently. P5 exhibited early-onset DI, whereas C7 manifested HL before DI. This discrepancy may reflect the absence of proactive DI screening in the historical group, potentially leading to a missing or delayed diagnosis of DI.

Additionally, two siblings in the newly screened cohort with the same frameshift mutation showed differing ages at diagnosis and clinical presentations, indicating possible intrafamilial variability. Similar discordance was also observed among siblings in the historical cohort, reinforcing the challenge of establishing genotype–phenotype correlations, even in genetically identical individuals.

Patients who screened positive on the polyuria/polydipsia questionnaire but tested negative for *WFS1* mutations did not develop overt WS1-related symptoms during the study period. Regular clinical, ophthalmological, and audiological assessments confirmed the absence of OA and HL in this group.

Tables 2–4 summarizes the clinical and genetic features of the newly genetically confirmed and historical WS1 patients. The age of diagnosis for DM was younger in the historical cases (5.1 ± 2.0 vs. 8.7 ± 3.4; *p*=0.04). There was no statistically significant difference between the ages for the diagnosis of WS1 (12.9 ± 5.0 vs. 9.6 ± 2.7; *p*=0.191), though the diagnostic delay from DM onset to WS1 diagnosis was significantly shorter in the screened group (median 1.8 vs. 6.9 years; *p* ≈ 0.015).

## 4. Discussion

In the present study, we implemented a brief polyuria/polydipsia questionnaire during follow-up visits for children with Type 1 DM, which enabled earlier identification of DI in several patients, before the onset of more classical WS1 features such as OA. These findings highlight the potential value of structured screening tools in uncovering subtle early manifestations of WS1.

In our cohort, the median age at WS1 diagnosis was 9 years and 6 months. In contrast, the meta-analysis by de Heredia et al. [10], which evaluated 412 patients, reported that OA typically appears at a median age of 11 years, while DI most commonly manifests around 13 years. In our study, using the questionnaire allowed DI to be identified earlier than OA in several patients, with a median DI diagnosis age of 9.3 years, remarkably earlier than those reported by de Heredia et al. [10]. This trend suggests that structured symptom-based screening may facilitate earlier recognition of DI than is typically reported in retrospective datasets.

In contrast, our historical WS1 cohort had a median age of 11 years for DI diagnosis, closely aligning with the Heredia timeline. The clinical sequence in these historical cases—DM followed by OA and then DI—mirrored the natural history described in the literature. This comparison emphasizes that proactive symptom screening, like our questionnaire, may effectively shift the diagnostic timeline and reveal WS1 earlier in its course.

Two siblings from the historical cohort (C3/F2 and C4/F2) also demonstrated an early presentation of DI, occurring after DM but notably before the appearance of OA and HL. This diagnostic sequence, though identified retrospectively, mirrors the clinical pattern observed in our newly diagnosed cohort. These cases further support the hypothesis that DI may precede other hallmark features of WS1 and emphasize the need for vigilant early symptom assessment, even in cases without structured screening protocols.

This discrepancy underscores the importance of early detection tools, such as the present polyuria/polydipsia questionnaire, for the timely identification of DI before more overt features like OA or HL develop. Our findings suggest that even a simple 5 min screening can support earlier recognition of WS1 features, enabling closer monitoring and appropriate management. This is particularly important given that DI in WS1 is often partial and initially difficult to diagnose [10]. A high index of suspicion is essential, and water deprivation testing should be considered in patients with DM who also present with visual, auditory, or neurological symptoms suggestive of WS1 [16]. Moreover, early diagnosis and intensive diabetes management can mitigate glucotoxicity, an established contributor of neurodegeneration in WS1, helping to delay progression to complications such as OA, HL, and neuropsychiatric symptoms [9, 13].

Among the six newly diagnosed patients, we identified seven *WFS1* variants: four missense, one in-frame duplication, and two frameshift mutations. Notably, two were novel: *WFS1* (NM_006005) c.2104G > T and c.1727_1744dup. The c.2104G > T variant, located in the topological domain, is scarce (gnomAD allele frequency: 6.201e-7, no homozygotes) and was confirmed to be in trans with the c.319G > A variant in a clinically diagnosed WS1 patient. In silico predictions support its pathogenicity (e.g., SIFT: 0, MetaLR: 0.95, FATHMM: −4.38). Similarly, the c.1727_1744dup variant, absent from population databases, affects the eighth transmembrane domain and alters protein length. It was also confirmed to be in trans with the p.Arg558Cys, a known pathogenic variant. Both variants localize to essential functional regions of the protein, and their segregation with characteristic WS1 features further supports their pathogenicity. However, functional studies are necessary to validate their impact precisely.

Our cohort demonstrates alignment with and deviation from published models when assessed in light of established genotype–phenotype classification systems. According to the de Heredia et al. [10] framework, three of our patients (5/5, 17/17, 18/17) were classified as Class A1, characterized by biallelic loss-of-function (frameshift) mutations. As expected, these individuals exhibited early-onset DM and DI, confirming the association between severe protein-truncating mutations and early multisystem involvement. Notably, two patients (17/17 and 18/17) harbored the identical homozygous c.2643_2644del mutation yet displayed markedly different ages of symptom onset, highlighting intrafamilial phenotypical variability.

Patients 3/3 and 10/10, carrying homozygous or compound heterozygous missense mutations, were classified as Class C and Group 2 under the Heredia and Rigoli systems [8, 10], respectively. While patient 10/10 presented with a mild and late-onset disease course, patient 3/3 developed DI, OA, and HL considerably earlier. This discrepancy underscores the limitations of genotype-based prediction models and points to the potential role of environmental or genetic modifiers. Patient 12/12, with a combination of missense and in-frame duplication mutations, fell into the intermediate Class B/Group 3 categories. Their clinical profile (including DI onset in adolescence and absence of OA) supports a partial loss-of-function mechanism, consistent with prior reports.

Two siblings from our historical cohort (C3/F2 and C4/F2) exhibited DI before OA and HL were analyzed alongside the newly diagnosed group. Using Figure 2, we compared variants from patients presenting initially with DI versus those with OA or HL to explore possible genotype–phenotype correlations. However, the distribution of variants across the *WFS1* gene appeared random, and no apparent mutational clustering could be associated with early DI onset.

Overall, our data support the general trends outlined in the genotype–phenotype models of Rigoli, Rohayem, and Heredia [8–10]. However, the considerable variability seen, particularly among patients sharing identical genotypes, reinforces the complexity of WS1 and the need for cautious interpretation of genetic findings. The early identification of DI in Class A1 cases further underscores the clinical utility of targeted screening tools in optimizing timely diagnosis and management.

This study has several limitations. First, the sample size is relatively small, limiting statistical power and generalizability. Second, functional validation was not performed for the novel variants detected, and pathogenicity assessments relied on in silico predictions and clinical correlation. Although functional studies were beyond the scope of this study, we recognize their critical role in variant interpretation and aim to explore opportunities for functional validation in future collaborative research. Third, although we primarily focused on patients presenting with DI symptoms, our protocol also included screening for other WS1-associated features; however, we did not perform universal *WFS1* genetic testing in all autoantibody-negative patients. This may have led to missed cases with subclinical or atypical presentations. Fourth, we did not apply broader monogenic diabetes gene panels, which may have identified alternative genetic causes of diabetes in our cohort. Finally, the absence of other WS1-related features (e.g., OA, HL) in some patients may reflect the relatively short follow-up period rather than the actual absence of disease progression. Complementary screening tools, such as optical coherence tomography (OCT) or hearing assessments, may also aid early detection in future studies.

In conclusion, the present study highlights that enhancing the diagnosis of WS1 by using a brief polyuria/polydipsia questionnaire as a simple yet effective tool for detecting DI in patients with WS1 may accelerate the diagnosis, enabling timely interventions and tailored follow-up strategies. Integrating targeted screening, comprehensive genetic testing, and vigilant clinical monitoring for early recognition of DI and diagnosis of WS1 is warranted. Expanding this approach to larger, antibody-negative diabetic populations may improve early detection and more favorable outcomes in WS1.

## Figures and Tables

**Table 1:** The questionnaire designed to evaluate symptoms of polyuria and polydipsia.

Question	Response
1. Can the polyuria or polydipsia be quantified	—
a. How much does your child drink per day?	—
b. How many times does your child urinate per day?	—
c. How much would they urinate each time?	—
2. What type of fluid does your child consume? (habitual polydipsia)	—
3. How long have these symptoms been present?	—
4. Does it interfere with normal activity?	—
5. Are there other associated symptoms such as polyphagia, vomiting, or altered mental status?	—

*Note:* Reference: [12].

**Table 2:** Genetic characteristics of newly identified and historical WS1 patients.

Patient	Variant^a^	Exon	Protein change	Protein domain	Zygosity	ACMG clas.	Publication (PMID)
P3/3	c.319G > A/ c.2104G > T	4/8	p.Gly107Arg/p.Gly702Cys	Topo/TM6	C-het	LP/LP	PMID:23103830/ Novel
P5/5	c.1523_1524del	8	p.Tyr508Cysfs^a^34	TM6	Hom	P	PMID:31850070
P10/10	c.376G > A	4	p.Ala126Thr	—	Hom	P	PMID:26773575
P12/12	c.1672C > T/c.1727_1744dup	8/8	p.Arg558Cys/p.Gly576_Gly581dup	−/TM8	C-het	P/VUS-LP	PMID:30014265/ Novel
P17/17	c.2643_2644del	8	p.Phe883Leufs^a^56	TM11	Hom	P	PMID:33879153
P18/17	c.2643_2644del	8	p.Phe883Leufs^a^56	TM11	Hom	P	PMID:33879153
C1/1	c.554G > A	5	p.(Trp185Ter)	—	Hom	LP	PMID:32938580
C2/1	c.554G > A	5	p.(Trp185Ter)	—	Hom	LP	PMID:32938580
C3/2	c.1885C > T	8	p.(Arg629Trp)	—	Hom	LP	PMID:27468121
C4/2	c.1885C > T	8	p.(Arg629Trp)	—	Hom	LP	PMID:27468121
C5/3	c.1532T > C	8	p.(Leu511Pro)	TM6	Hom	VUS/LP	PMID:21968327
C6/4	c.2416G > C	8	p.(Ala806Pro)	Topo	Hom	LP	PMID:24890733
C7/5	c.1523_1524del/c.1385_1393del	8/8	p.(Tyr508Cysfs^a^34)/p.(Glu462_Thr464del)	−/TM6	C-het	P/LP	PMID:24890733 PMID:20738327

*Note:* Protein Domain abbreviations: Topo, topological domain; TM, transmembrane domain (number = transmembrane segment). Zygosity: C-het, compound heterozygous; Hom, homozygous. ACMG Clas.: variant classification according to ACMG/AMP guidelines.

Abbreviations: LP, likely pathogenic; P, pathogenic; VUS-LP, variant of uncertain significance/likely pathogenic.

*⁣* ^a^Variants are described according to NM_006005 (RefSeq transcript).

**Table 3:** Literature-based variant classifications of newly identified and historical WS1 patients.

Patient	Rigoli et al. [8] group	Rohayem et al. [9] classification	de Heredia et al. [10] classification
P3/3	Group 2	Type III/Type III	Class C
P5/5	Group 1	Type I	Class A1
P10/10	Group 2	Type III	Class C
P12/12	Group 3	Type III/Type II	Class B
P17/17	Group 1	Type I	Class A1
P18/17	Group 1	Type I	Class A1
C1/1	Group 1	Type I	Class A1
C2/1	Group 1	Type I	Class A1
C3/2	Group 2	Type III	Class C
C4/2	Group 2	Type III	Class C
C5/3	Group 2	Type III	Class C
C6/4	Group 2	Type III	Class C
C7/5	Group 3	Type I/Type II	Class A2

**Table 4:** Clinical characteristics of newly identified and historical WS1 patients.

Patient	Age at WS1 diagnosis	Gender	BMI (percentile, SD)	Height (percentile, SD)	Age at DM onset	Age at DI onset	Age at OA onset	Age at HL onset
P3/3	7 year 1 month	Female	15.1 (40.5 p, −0.24)	118.4 cm (72.9 p, −0.89 SD)	6 year	6 year 10 month	11 year 2 month	8 year

P5/5	7 year 7 month	Female	20.3 (87.9 p, 1.17 SD)	149.2 cm (97.8 p, 2.01 SD)	5 year	7 yearr 4 month	11 year 6 month	None

P10/10	17 year	Female	22.4 (60.6 p, 0.27 SD)	155.5 cm (9.8 p, −1.29 SD)	13 year	16 year 9 month	None	None

P12/12	16 year	Male	18.5 (2 p, −2.05 SD)	181.5 cm (80 p, 0.86 SD)	12 year	15 year 9 month	None	None

P17/17	6 year 2 month	Male	16 (63.7 p, 0.35 SD)	118.6 cm (62.5 p, 0.32 SD)	5 year 11 month	5 year 11 month	None	None

P18/17	11 year 5 month	Female	17.8 (14.7 p, −1.05SD)	151.2 cm (9.5 p, −1.31SD)	11 year	11 year 2 month	12 year 11 month	None

C1/1	20 year	Male	23.2 (51.9 p, 0.05 SD)	176 (48.8 p, −0.03 SD)	2 year	20 year	None	2 year

C2/1	20 year	Male	24.3 (65.5 p, 0.4SD)	179 (67.3 p, 0.45 SD)	5 year	None	None	2 year

C3/2	8 year	Female	15.8 (49.2 p, −0.02 SD)	124 (30.5 p, −0.51 SD)	6 year	8 year	9 year	11 year

C4/2	12 year	Female	19.0 (51.2 p, 0.03 SD)	148 (21.4 p, −0.79 SD)	4 year	12 year	16 year	14 year

C5/3	11 year	Female	20.7 (81.3 p, 0.89 SD)	146 (53.5 p, 0.09 SD)	8 year	11 year	10 year	12 year

C6/4	9 year	Male	16.7 (55.5 p, 0.14 SD)	131.2 (43.6 p, −0.16 SD)	7 year	9 year	12 year	None

C7/5	10 year 11 month	Female	21.4 (85.5 p, 1.06 SD)	138 (11.9 p, −1.18 SD)	4 year	10 year	None	6 year

Abbreviations: DI, diabetes insipidus; HL, hearing loss; OA, optic atrophy; SD, standard deviation.

## Data Availability

The data that support the findings of this study are available upon request from the corresponding author. The data are not publicly available due to privacy or ethical restrictions.
